# Care and Treatment of the Six Year Old Molars

**Published:** 1868-01

**Authors:** R. G. Snow


### CARE AND TREATMENT OF THE SIX YEAR MOLARS.

BY R. G. SNOW.

Read before the Buffalo Dental Association, December 2, 1867.

MR. PRESIDENT AND GENTLEMEN: In treating this subject this evening, I shall not attempt anything elaborate or exhaustive; but rather endeavor to throw out a few hints, in hope of inducing others to set forth their views, so as to elicit, I hope, a useful and profitable discussion.

In the care of the teeth of children, it is very desirable to preserve every tooth in as sound and healthy a condition as possible: and from their predisposition to decay, the first or six year molars, perhaps require more careful attention than any of the others. But by early and proper treatment they may in general be preserved to old age.

These teeth are formed and show themselves through the gums in early childhood, while the vital forces are in general not yet fully developed, and when the processes of ossification and enamelization are liable to be interrupted and encroached upon by the diseases incident to that age. And therefore, when they are first protruded through the gums, they are less dense and hard than the teeth formed later in life, and of course more liable to the early attacks of disease.

In the formation of the various bones of the body, there are one or more points where ossification begins, and from which it radiates until the bone is completely formed. The same law holds good in the formation of the teeth. And in the process of enamelization of these molar teeth, the process begins on the corners or four highest points on the lower ones, and at the four or five or more prominent points on the upper ones. From the points of commencement they gradually radiate towards each other and over the body of the tooth, until they meet in the centre of the crown; and when the vital forces are strong, and the process carried on to perfection, unite, and form a perfect covering and protection for all the exposed part of the tooth. But from sickness, or from natural delicacy of constitution, or any other cause that weakens the vital forces, this union of the enamel in the centre of the crown in many cases, fails to take place. An early examination with a fine instrument will often reveal this fact, even before decay has commenced at this point. The bone or dentine being thus exposed to the action of external agents, decay is almost always found at this point, I may say always when this perfect union of the enamel has failed to take place.

Now in the care of these teeth, examination should be frequently made and the teeth carefully filled with gold on the first appearance of decay.

A great amount of ignorance exists among parents and even among physicians in regard to these teeth; many, I may say most parents looking upon them as deciduous teeth; and therefore their preservation a matter of little consequence. From this cause alone thousands of these teeth are neglected, and allowed to decay so far that they are past remedy and must be extracted.

One of the first duties of the Dentist is to instruct parents in the care of these teeth, and impressing upon them the necessity and importance of carefully watching them, and having them properly filled on the first indication of decay.

This should never be omitted when a child is presented for an examination of its deciduous or other teeth. Indeed, I think it proper for the Dentist to improve every possible opportunity to give a short lecture on dentition. You will hardly fail on any occasion to find auditors that need instruction.

Now as I said before, these teeth are as easily preserved as any of the others if they are brought under our care in an early stage of the disease; but from carelessness or the lamentable ignorance just mentioned, it often happens that the little patient is brought to the Dentist with the tooth aching from an exposed nerve; and now arises the important question, what shall be the treatment? And on this there is a good deal of difference of opinion among Dentists. I think the majority of Dentists in cases of this kind advise extraction at once without any effort to save the tooth. This of course puts a quietus on the matter, and saves a good deal of trouble both to the Dentist and the patient. But is it the best practice? Is it best for the interest and well being of the little patient? Very doubtful.

We all condemn the practice of extracting the deciduous teeth prematurely, because we say it prevents the regular and proper growth and development of the jaw. Does not the same principle hold good as to the extraction of these six year molars? Is not the jaw now in a state of growth and development? And does not the extraction of a large molar tooth have as much effect in arresting this growth and development as the early loss of a deciduous tooth?

In the *Dental Cosmos* for May, 1866, is an article from the pen of Dr. A. C. Hawes, of New York, in which he takes the ground that the six year molars must never be extracted during the period of childhood while the jaw is growing; or at any rate not until all the permanent teeth anterior to these are well through the gums and properly in place. He contends that the early extraction of these teeth favors, instead of preventing irregularity; that it tends to produce a narrow-pointed jaw, a rabbit-mouthed appearance in the patient. This is certainly philosophical, and undoubtedly true, as by the arrested development, produced by this early extraction, you have the narrow, undeveloped jaw of the child still remaining, after your patient has attained adult age.

The fact does not seem to be recognized by many who practice our profession, that the presence of the teeth are necessary to the proper and perfect growth and development of the jaw, and that it is absolutely and forever prevented by their premature extraction.

The same writer contends that American women, particularly among the wealthier classes, are coming to be noted for the narrowness of the lower part of the face; and he attributes it in a great majority of cases to the early extraction of the six year molars, or perhaps a bicuspid on each side to prevent or cure irregularity.

He goes further, and says, that in cases of irregularity from a crowded condition of the teeth, no tooth must be extracted to make room for the others; at least, the case must be extreme that requires it. The practice of extracting a bicuspid on each side in cases of this kind he condemns most pointedly; and this, although it is recommended by nearly all, and I think I may say all authors I ever read on the subject.

His plan is to enlarge the circle of the jaw so as to make room for the teeth. This may cause considerable trouble both to the Dentist and the patient, but when persevered in and properly done, you secure to your patient a better development; a better expression of countenance and a better set of teeth than by the old plan of extraction.

Perhaps you will say that these views are a little extreme, but I think they are no more so than truth requires, and that they are entitled to a most careful consideration. Our practice in this respect needs reforming and improving. We are all of us too ready and too willing to extract teeth.

I cannot but look upon it as a serious misfortune for a child to lose one of these teeth. I lately saw a case which strikingly exemplified its effect. It was in a middle aged man, who had a very good set of teeth; but in early boyhood he had the misfortune to lose the right lower first molar, and also the adjoining second bicuspid; leaving quite a large vacant space on that side of the lower jaw. The consequence was that the first bicuspid and canine had settled back considerably, and the incisors had settled off to the right as much or more than the width of a tooth. The left canine was much more prominent than the right, destroying the symmetry in the appearance of his mouth, and almost amounting to deformity.

What I have already said will perhaps sufficiently indicate the treatment I would recommend. I would use every possible means to preserve the tooth, and only resort to extraction when all curative treatment had failed. I know it is considered more difficult to destroy the nerve and fill the roots successfully in the tooth of a child, than in that of an adult; still, by careful treatment and patience, it may in the majority of instances be done. If you can preserve the tooth till the jaw has acquired its growth, you have accomplished a great deal, and done much more towards securing to your patient a well developed denture, and a pleasing expression of countenance than can be accomplished by the old method of early extraction.

I know that many cases occur, in which from the carelessness and negligence of parents, the Dentist is unable to do what is best for his little patient. When such cases come before you I would recommend the careful application of the proper remedy to destroy the vitality of the nerve, and let the tooth remain to gradually decay away; and only extract it at the eleventh or twelfth year, when the second molars are about to make their appearance. Even in this way, the tooth may assist in some degree in securing to your patient a proper and normal development. The loss of a tooth later in life is of little consequence to what it is in early childhood.

